# Identifying the Enhanced Oil Recovery Polymer in Sludge Formed in Water Lines

**DOI:** 10.1021/acsomega.6c00158

**Published:** 2026-02-28

**Authors:** Gustavo G. Celestino, Júlia V. Nunes, Elizabete F. Lucas

**Affiliations:** a Programa de Engenharia Metalúrgica e de Materiais/COPPE/LADPOL, 28125 Universidade Federal do Rio de Janeiro, Av. Horácio Macedo, 2030, bloco F, Rio de Janeiro, RJ 21941598, Brazil; b Instituto de Macromoléculas/LMCP, Universidade Federal do Rio de Janeiro, Rua Moniz Aragão, 360, bloco 8G/CT2, Rio de Janeiro, RJ 21941594, Brazil

## Abstract

Sludge formation is extremely undesirable in the petroleum industry. Such sludge formation can be induced by different causes. The deposition of asphaltenes, waxes, inorganic salts, and even corrosion products is among the main causes of sludge formation. Therefore, it is very important to characterize the sludge to identify the causes of its formation and apply procedures to minimize or even avoid it. A protocol to characterize sludge in oil and water lines is already available. However, the role of the polymer used in enhanced oil recovery (EOR) on the sludge formation is already unknown. In this work, a procedure to identify the presence of the EOR polymer in sludge samples was developed. Two techniques to detect polymers were selected: size-exclusion chromatography (SEC) and multiangle light scattering (MALS). These techniques were validated by using polymer solutions at different concentrations. Then, synthetic sludge samples were prepared based on an emulsion of waxes and asphaltenes in toluene and brine, without and with different concentrations of polymers. The synthetic sludge samples were submitted to successive extractions with organic solvents to recover the residue, which was dispersed in water, filtered, and analyzed by SEC and MALS. The procedure was validated with synthetic sludge and applied to eight real sludge samples coming from a Brazilian oil field. The polymer was detected in all of them, and its concentration could be estimated. This kind of characterization makes it possible to identify the causes of sludge formation, preventing it and helping to reduce the environmental impact of the oil industry.

## Graphical abstract

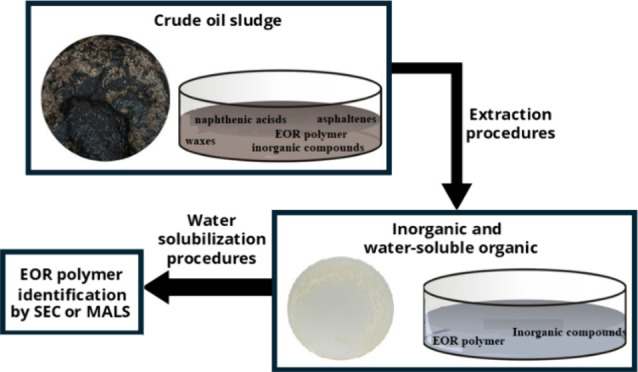

## 1 Introduction

Enhanced oil recovery (EOR) is used when secondary oil recovery does not become effective. This method is based on the injection of aqueous solutions with a large number of chemicals to improve the oil recovery. Usually, polymers with very high molar mass are used in this method with concentrations between 500 and 2000 ppm because these compounds generate solutions with higher viscosity, having as its main characteristics the decrease in mobility and, at the same time, the decrease in the water relative permeability compared to crude oil.^1,2^ Therefore, the presence of this chemical increases the oil sweep efficiency. There are some studies to use biopolymers, such as xanthan and guar gum during this process, but the most common polymer used is synthetic, based on polyacrylamide (PAM).^3–5^

The EOR process is very efficient to increase petroleum production; nonetheless, this process can bring consequences for the following steps, especially in flocculation of produced water. Dalmazzone et al.^6^ and Ferraz et al.^7^ studied the influence of HPAM on demulsification and quality of water through the total oil grease (TOG) method. The researchers observed that the presence of HPAM slightly decreased the emulsion stability; however, the water quality also decreased after the determination of TOG. The increase in viscosity of the water phase can justify the decrease in water quality because it allowed that the oil drop was dispersed in the water phase. Sjöblom et al.^8^ studied the w/o emulsion stability in the presence of HPAM as well as Ferraz et al.,^7^ and the authors did not observe the influence of HPAM on emulsion stability in the absence of a demulsifier. On the other hand, the authors also observed that the combination of HPAM and a demulsifier decreased the emulsion stability. Li et al.^9^ observed the same phenomenon observed by Ferraz et al.^7^ and Dalmazzone et al.;^6^ however, the justified high stabilities of o/w emulsion in the presence of HPAM was observed using zeta potential, and this measure indicated that HPAM stabilizes the o/w emulsions due to lower values of potential with the increase in the HPAM concentration. Another possible consequence is sludge formation due to chemical incompatibility. There is a report that the presence of PAM during EOR can be causing sludge formation because of the reaction between PAM and the cationic coagulant.^10^

Sludge formation is related to flow assurance issues, as well as organic and inorganic deposition, corrosion, and emulsion.^11–17^ Sludges can be considered a complex emulsion, being able to contain water, oil, and/or inorganic solid particles.^18,19^ The characterization of these sludges is very important to prevent their formation, and the solvent extraction is used to determine the composition of sludges.^20–22^ Celestino et al.^23^ presented a protocol for determining precisely the compound classes that induce the sludge formation: aromatic, aliphatic, more polar, and residue. In addition, complementary analysis was used to determine the identification of waxes in the aliphatic fraction and the kind of inorganic compound observed in the residue. After an exhaustive literature search on the quantification of polymers in sludge, only Chen et al.^24^ have proposed to identify and quantify EOR polymers in the sludge. The authors used the Soxhlet extraction process to separate the oil phase of the sludge using a mixture of petroleum ether, benzene, ethanol, and chloroform (1:1:1:1, in volume), and the water-soluble polymer content was separated by putting 5 g of suspended solids in distilled water. Both percentages were determined by gravimetry after solvent evaporation. In that method, the mass value obtained by gravimetry indicates not only the mass of PAM compounds but also all water-soluble compounds such as different types of salts or other polymers. Although this method can be used to determine the water-soluble compounds, this technique is not precise to determine the presence of the EOR polymer in sludges.

To identify and quantify polymers, there are some methods for determining both their chemical structure and their molar mass. Spectroscopy techniques, such as nuclear resonance magnetic (NMR) and Fourier transform infrared (FTIR), are used to determine the chemical structure of the compounds.^25^ These techniques may be used to characterize the polymer; however, any water-soluble organic compound present in the sludge may be an interferent. Membrane osmometry, ultracentrifugation, matrix-assisted laser desorption/ionization–time of flight (MALDI-TOF), size-exclusion chromatography (SEC), and multiangle light scattering (MALS) are examples of techniques for determining high molar mass. Membrane osmometry has limitations, such as long equilibration times and temperature sensitivity; MALDI-TOF is expensive and requires an ionizable matrix; and ultracentrifugation is less applicable because it requires a pure sample. Thus, SEC and MALS are applicable to polymer identification because they are chromatographic and light scattering methods, respectively. SEC allows for the separation of components, ensuring that other water-soluble molecules do not interfere with the analyses, and MALS identifies the light scattering caused by the polymer.^26^

Identifying and quantifying PAMs in sludge is more challenging than identifying polymers in the aqueous phase, as it requires the development of a replicable extraction method prior to the analytical procedure. To the best of our knowledge, there is no reliable procedure to identify the presence of PAM in the sludge composition. Therefore, the aim of this study was to establish a methodology to identify EOR polymers in real sludge samples produced in a Brazilian oil field. Due to the high chemical complexity of sludge, whose composition (inorganic salts, asphaltenes, corrosion products, and waxes, among others) can interfere with extraction and filtration, as well as chromatographic analyses, the strategy used was as follows: (i) select a technique to detect polymers among other compounds in a sludge, (ii) validate the technique with polymer solutions at different concentrations; (ii) prepare synthetic sludge samples without and with different concentrations of EOR polymers, (iii) submit the synthetic samples to the successive extractions with organic solvents, (iv) recover the water-soluble compounds from the residue remaining at the end of the successive extractions and analyze them by SEC and MALS techniques, and (v) apply the new procedure to the real sludge samples.

## 2 Experimental Section

### 2.1 Materials

Seven sludge samples from a Brazilian oil field were donated by Equinor Brasil, named WPL01, WPL02, WPL03, WPL04, WPL05, WPL06, and WPL07. Toluene (CAS 108-88-3, 99%), *n*-heptane (CAS 142-82-5, 99%), and dichloromethane (CAS 75-09-2, 99%) were purchased from Isofar, Duque de Caxias, Brazil. Asphaltenes C7I were previously extracted from a Brazilian crude oil.^11^ Commercial waxes (CAS 8002-74-2, 95%), melting point range 56–58 °C, were supplied by Sigma-Aldrich, São Paulo, Brazil. The following salts, supplied by Isofar, Duque de Caxias, Brazil, were used to prepare the brine: sodium chloride (CAS 7647-14-5, P.A., NaCl), sodium bicarbonate (CAS 144-55-8, P.A., NaHCO_3_), potassium chloride (CAS 7447-40-7, P.A., KCl), magnesium chloride hexahydrate (CAS 7791-18-6, P.A., MgCl_2_·6H_2_O), calcium chloride dehydrate (CAS 10043-52-4, P.A., CaCl_2_·2H_2_O), strontium chloride hexahydrate (CAS 10025-70-4, P.A., SrCl_2_·6H_2_O), barium chloride dihydrate (CAS 10361-37-2, P.A., BaCl_2_·2H_2_O), silicon oxide (CAS 7631-86-9, P.A., SiO_2_), and acetic acid (CAS 64-19-7, 99%, CH_3_COOH). Polyacrylamide (PAM) (CAS 9003-05-8, 96%)-FLOPAAM AN125/Floerger^5^-was supplied by Equinor Brasil.

### 2.2 The Development Strategy

The first step was to select the same sample of polymer (PAM) that is used in EOR operations in the petroleum field from where the real sludges were withdrawn. Since the sludges can be constituted of different components, the polymer, if present, needs to be separated and detected. The following strategy was used in this work:

(1) Selection of two techniques able to identify the polymer even though they are mixed with molecules of low molar mass. In this step, size-exclusion chromatography (SEC) and dynamic light scattering (MALS) were selected to identify the presence of the PAM in the sludges.
(2) Obtaining the responses of the SEC and MALS techniques for the PAM sample solubilized in brine (with a composition like that of the field under study) in a concentration range of 5–2000 ppm. The concentration of 5 ppm was used to verify the method’s sensibility to low polymer contents, and the maximum concentration was established at 2000 ppm because concentrations above this value are not found in produced water samples.
(3) Production of synthetic sludges containing PAM at different concentrations (from 100 to 2000 ppm).
(4) Application to synthetic sludges the same extraction procedure applied to real sludges,^23^ to separate organic fractions from water-soluble materials, in which polymers might be present.
(5) Utilization of the SEC and MALS techniques to water-soluble extracts to confirm the polymer detection that was added to synthetic sludges to validate the polymer identification’s method.
(6) Application of these methods to detect polymers in sludges that were withdrawn during the oil production.

### 2.3 Methodologies

#### 2.3.1 Preparation of Polymer Solutions in Brine

First, brine containing 91,065 ppm of a mixture of salts was prepared according to Table 1. Then, a polymer was added at different concentrations (from 5 to 2000 ppm). The polymer solutions were filtered through a 0.45 μm membrane. At the end, the solutions were injected in SEC and MALS to determine the retention time/signal intensity and the signal intensity, respectively.

**Table 1:** Brine Composition

**ion**	**salts**	**concentration of salts**
Na^+^	NaCl	80,800
Ca^2+^	CaCl_2_	6440
HCO_3_^–^	NaHCO_3_	200
Mg^2+^	MgCl_2_	2030
K^+^	KCl	810
Sr^2+^	SrCl_2_	430
Ba^2+^	BaCl_2_	30
	SiO_2_	25
	CH_3_COOH	300
Total concentration		91,065

#### 2.3.2 Preparation of Synthetic Sludge Samples without and with PAM

Six synthetic sludge samples were prepared. The preparation procedure was based on emulsion formation, followed by solvent evaporation. Therefore, two phases were prepared: an aqueous phase and an oily phase. The aqueous phase was constituted of brine (∼91,065 ppm of a mixture of salts), and the oily phase was prepared with 5 w/v% of asphaltenes and 5 w/v% of waxes in toluene. The phases were mixed for 1 min with a glass rod, followed by mechanical stirring (Polytron) for 6 min at room temperature. This emulsion was put in an oven at 90 °C for 48 h to evaporate the solvents. This represents the sludge without a polymer, which was prepared to be used as a reference. The same procedure was used to prepare the synthetic sludge containing a polymer, which was added to the aqueous phase, before emulsion formation, at the following concentrations: 100, 250, 500, 1000, and 2000.

#### 2.3.3 Successive Extractions of Synthetic Sludge Using Solvents with Different Polarities

The synthetic sludge samples were submitted to the successive extractions step, which followed the procedure previously described by Celestino et al.^23^ The sludge was placed in a cellulose cartridge that was placed in the Soxhlet extractor. Initially, the organic compounds were extracted using a sequence of solvents with different polarities: *n*-heptane, to extract the aliphatic compounds, followed by toluene, to extract the aromatic compounds, and dichloromethane, to extract more polar compounds. After that, the cellulose cartridge was removed from the extractor, and the residue was recovered. This procedure provides the quantification of the aliphatic, aromatic, more polar compounds, and residue fractions, as shown in Table 2.

**Table 2:** Solvents Used in the Successive Extraction and Respective Isolated Fractions

**solvent**	**isolated fraction**
*n*-heptane	aliphatic compounds
toluene	aromatic compounds
dichloromethane	more polar compounds
residue	

#### 2.3.4 Procedure to Recover Water-Soluble Compounds from the Residue

The residue obtained after the successive extractions was dispersed in water to solubilize the water-soluble compounds and, consequently, separate them from the water-insoluble compounds. Afterward, the system was vacuum-filtered using a cellulose membrane (porosity 2.2 μm), and the aqueous phase was analyzed by SEC and MALS. This procedure was applied to the residues coming from the synthetic and real sludges previously obtained.^23^ To observe the appearance of water-soluble compounds, after vacuum filtration, the supernatant of the synthetic sludges was collected in a Kitassato and put into an oven at 95 °C for 24 h.

#### 2.3.5 Analysis of the Aqueous Phase Using SEC and MALS

These procedures were applied to polymer solutions, and the aqueous phases were recovered from the synthetic and real sludges.

##### 2.3.5.1 Size-Exclusion Chromatography (SEC)

SEC was selected to identify the presence of the polymer (used in EOR operations) because it can separate the molecules as a function of their molar mass. Since the polymer presents much higher molar mass than the other possible molecules in the sludge composition, this technique was considered suitable. An Agilent Technologies 1260 infinity size-exclusion chromatograph with multiangle light scattering as a detector (SEC-MALS), using a SHODEX SB-806 HQ column and sodium sulfate 0.2 mol/L as the mobile phase, was used. The equipment was previously calibrated with poly­(ethylene oxide) standards.

##### 2.3.5.2 Multiangle Light Scattering (MALS)

MALS was selected because it can determine the weight-average molar mass. Because the EOR polymer presents very high molar mass, it is possible to analyze its molar mass and take it as a reference when analyzing the unknown samples. A Wyatt model Dann Heless II multiangle light scattering was used in this study. Initially, the brine without PAM was injected into the equipment to determine the baseline position. After that, the PAM solutions were injected into the equipment from lower to higher concentrations. At the end, brine was injected into the equipment again to confirm the baseline position.

Figure 1 presents a schematic representation of the complete extraction and quantification procedure.

**Figure 1:**
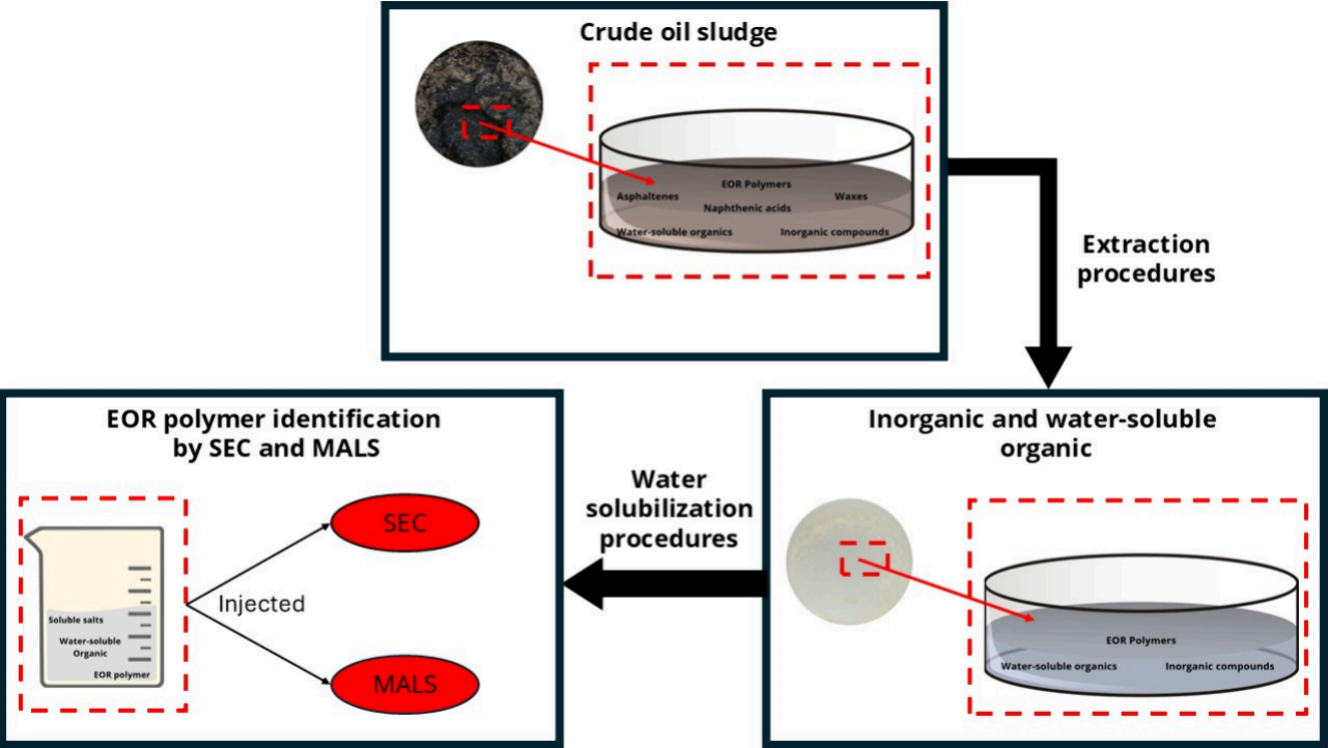
Schematic representation of the complete extraction and quantification procedure.

## 3 Results and Discussion

### 3.1 Analysis of Polymer Solution Using SEC and MALS

The selection of a technique to identify the presence of PAM was based on the fact that the EOR polymer presents a molar mass significantly higher than any other water-soluble compound in the sludge. For this study, we selected a PAM used in EOR operations that presents a molar mass around 10,000,000 g/mol. Therefore, SEC was the first option for this purpose because this technique allows the separation of the compounds based on its hydrodynamic volume, making it possible to separate PAM from the relative low-molar-mass water-soluble organic compounds that constitute the real sludge. Analyzing the sample directly in a light scattering device, which can determine weight-average molar masses of polymers, is an alternative method to identify the presence of polymers.

First, it was necessary to establish the standard response of both techniques at the used conditions for aqueous solutions of polymers. The polymer was analyzed at a concentration range from 5 to 2000 ppm in brine (∼90,000 ppm of a salt mixture).

#### 3.1.1 Size-Exclusion Chromatography (SEC)

Figure 2a shows the peaks obtained for each polymer concentration. As expected, the higher the polymer concentration, the larger the peak area. At this time, the retention times, between 17 and 28 min, for the polymer sample was established, using the mentioned equipment at the analysis conditions described in the Experimental Section. The limit of detection (LOD) and limit of quantification (LOQ) were calculated according to eqs 1 and 2, respectively.

$$\mathrm{LOD}=\frac{3.3\times \sigma }{S}$$ 1

$$\mathrm{LOQ}=\frac{10\times \sigma }{S}$$ 2

where *S* is the slope of the calibration curve and σ is the standard deviation of the integration of the analyses carried out without PAM.

**Figure 2:**
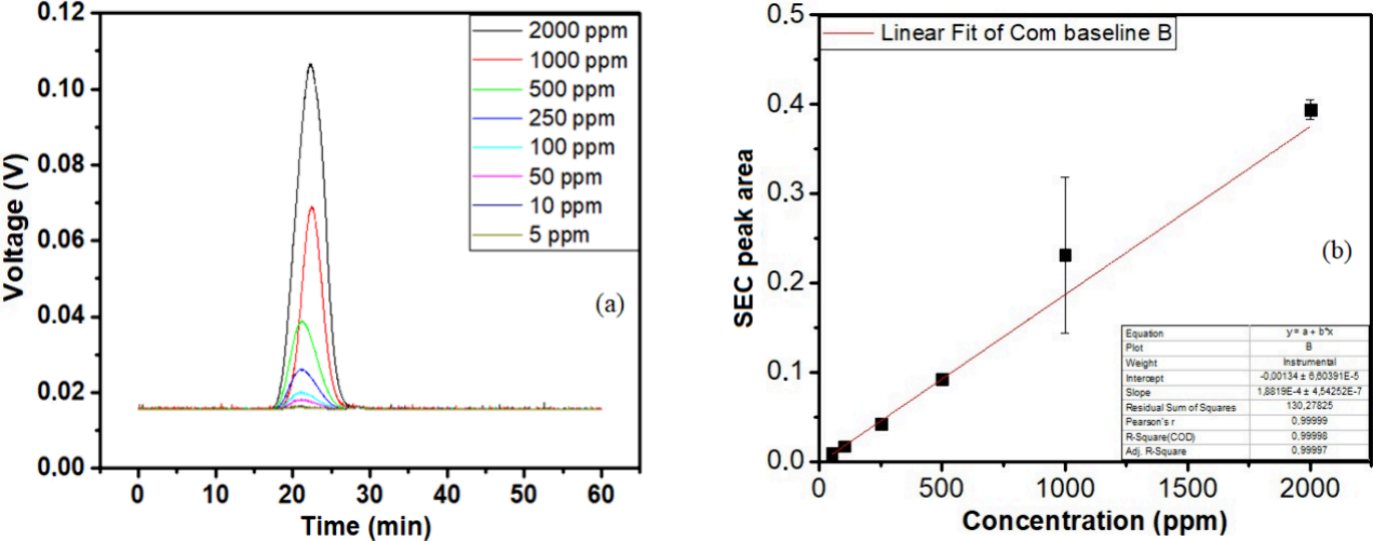
(a) SEC chromatograms of PAM from 5 to 2000 ppm. (b) Curve of the peak area as a function of polymer concentration in brine.

Figure 2b shows the linear regression of the integration of the peak area against the polymer theoretical concentration, with the equation *y* = 1.8819 × 10^–4^ *x* – 0.00134, with the correlation coefficient of 0.9999. The results obtained for 5 and 10 ppm PAM were excluded because their signals were close to the baseline one. In addition, LOD and LOQ were beyond these values, being 5 and 15 ppm, respectively Therefore, the method can identify a polymer and estimate its concentration from 50 ppm in the aqueous solution injected in the SEC, using the analytical curve exhibited in Figure 2b. The minimum detection limit was determined by extrapolating the curve to lower concentrations.

#### 3.1.2 Multiangle Light Scattering (MALS)

Figure 3 shows the signal intensities for all dispersions. The presence of a polymer was confirmed by the weight-average molar masses determined by the equipment, which was around 10^7^ g/mol for all samples containing PAM. This means that the technique can detect the polymer at concentrations as low as 10 ppm. As expected, the intensity of the signal increased with increasing polymer concentration in brine.

**Figure 3:**
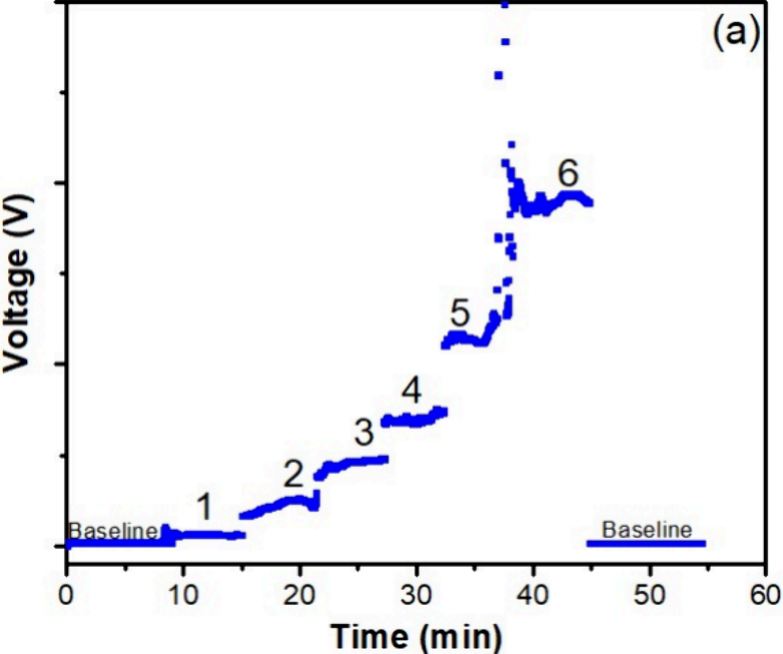
MALS signals of the aqueous solution of PAM in brine at different concentrations: (1) 10, (2) 50, (3) 250, (4) 500, (5) 1000, and (6) 2000 ppm.

These techniques were chosen because they demonstrated high efficiency in detecting polymers using signals obtained by the equipment. Due to the small amount of polymer solution relative to the mobile phase, the salt concentration in the solution does not interfere with the analyses. Regarding the interference of other polymers possibly present in the medium, this can be mitigated by modifying the ionic strength of the mobile phase and/or by selecting the most suitable separation column, allowing for more selective separation with respect to molar mass. For this reason, it is always necessary to perform preliminary analyses with a standard of the target polymer to determine the retention time.

### 3.2 The Synthetic Sludge Samples

The synthetic sludge samples were produced with the aim of investigating (1) the possible influence of the water-soluble organic compounds on the identification of the PAM and (2) the detection limit of the PAM when using the sludges prepared at different PAM concentrations. This strategy was chosen due to the high complexity of the composition of real sludge, and some constituents could interfere during the development of the methodology.

Figure 4 shows the synthetic sludge without and with 1000 ppm PAM. The synthetic sludges presented different aspects after the solvent evaporation. It was observed, for the synthetic sludge prepared without PAM, that phase separation occurred when the emulsion was taken from the oven because the system was not well-dispersed. This was not observed for the synthetic sludges that were prepared at 1000 and 2000 ppm PAM, probably due to the increase in the viscosity provoked by PAM in the emulsion. The synthetic sludge prepared with 1000 ppm PAM was well-dispersed, and some phase separation was observed during solvent evaporation. The high viscosity caused by the PAM high concentration (10,000 ppm) made the coalescence of the organic phase difficult while the solvents were evaporated; therefore, we observed a more dispersed sludge.

**Figure 4:**
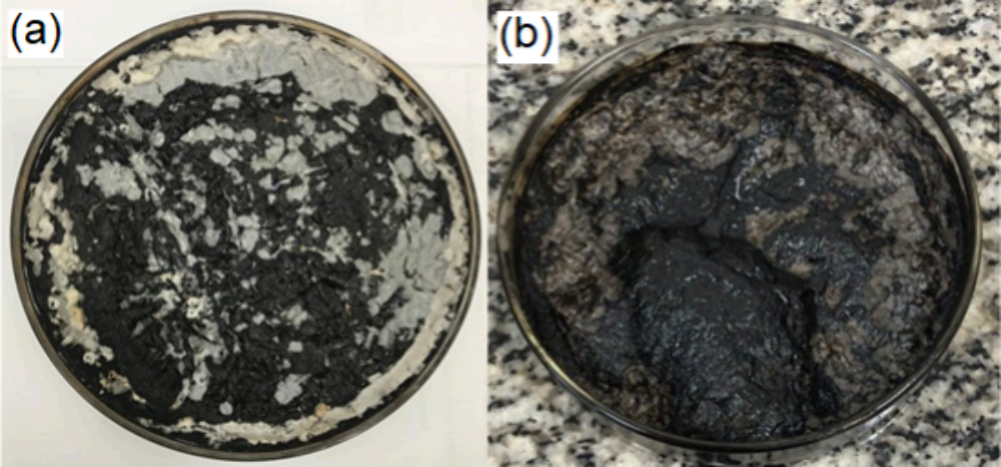
Aspect of the synthetic sludge containing wax, asphaltenes, and salts, after being heated in the oven at 95 °C for 24 h: (a) without PAM and (b) with 1000 ppm PAM.

After being submitted to successive extractions, the residue presented the appearance shown in Figure 5a. All solid waste presented a dark coloring. Probably some organic compounds were still retained in these solids such as asphaltenes. This also happens with real sludge,^23^ which can retain in the residue some organic compounds, besides the inorganic compounds. After the residue was dispersed in water and the solution filtered, the water-soluble compounds could be recovered. Figures 5b and 5c show, respectively, the membrane with water-insoluble compounds retained on it and the solid containing only water-soluble compounds. Most likely, a real sludge contains inorganic salts, PAM, and/or some other water-soluble compounds. However, it can be expected that the white solid, obtained after synthetic sludge extractions, contains only inorganic salts and PAM since these were the water-soluble compounds used to prepare the sludge.

**Figure 5:**
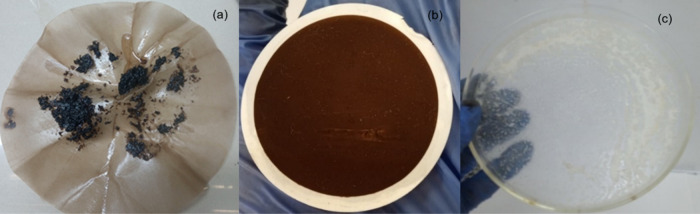
Material recovered after filtrating the residue dispersed in water: (a) solid waste obtained after extracting the no water-soluble compounds from the sludge prepared to 1000 ppm PAM, (b) membrane with water-insoluble compounds retained on it, and (c) water-soluble compounds, after evaporating the solvent.

### 3.3 Analyses of the Water-Soluble Compounds Obtained by the Residue of the Synthetic Sludge Samples

After the water-soluble compounds were isolated, the next step was related to their analysis by the selected techniques: SEC and MALS.

#### 3.3.1 Size-Exclusion Chromatography (SEC)

Figure 6 shows the chromatograms of the water-soluble compounds recovered from the synthetic sludges prepared without PAM and with 2000 ppm PAM. The polymer was detected exactly at the same retention time range as that observed in Figure 2a, confirming the presence of PAM, and no signal was observed for the water-soluble sample recovered from the synthetic sludge without PAM. The peak areas obtained for the samples coming from the synthetic sludges were converted in estimated concentrations using the equation exhibited in Figure 2b (*y* = 1.8819 × 10^–4^ *x* – 0.00134). The results are shown in Table 3. The estimated concentrations were lower than the theoretical ones in the composition of the synthetic sludges. This can indicate that material was lost during the procedure, which includes successive extractions, solubilization in water, and filtration through the membrane. Therefore, the concentration determined for PAM is not so reliable due to procedure losses and because the methodology was not quantitative. However, the aim of the project was achieved, which was to identify the presence of a polymer in all sludges prepared with a polymer at different concentrations.

**Figure 6:**
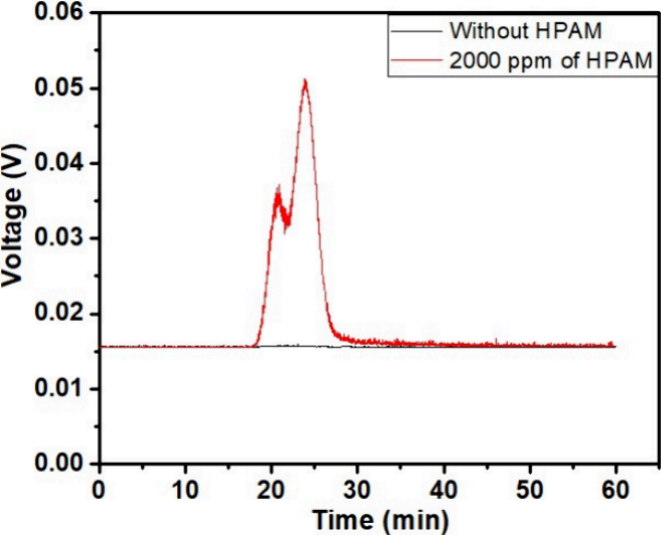
SEC chromatograms of the aqueous solution obtained from the residue of the synthetic sludge without PAM and with 2000 ppm PAM.

**Table 3:** Estimated PAM Concentration after Successive Extractions and Isolation of the Water-Soluble Compounds

**synthetic sludge**	**theoretical PAM concentration (ppm)**	**estimated PAM concentration (ppm)**
1	0	0.0
2	100	40.74 ± 0.43
3	250	59.16 ± 1.41
4	500	79.74 ± 2.60
5	1000	753.55 ± 196.41
6	2000	785.18 ± 11.88

The cause of the high losses may be related to the polymer degradation during shear stress when synthetic sludge is used. For this reason, the methods used can only be considered qualitative to semiquantitative.^27^

#### 3.3.2 Multiangle Light Scattering (MALS)

Figure 7 shows the MALS signals of water-soluble compounds recovered from the synthetic sludge samples containing 0, 100, and 1000 ppm PAM. The polymer identification was related to the high molar mass (∼10^7^ g/mol) detected by the analyses. The signal intensities are related to the concentrations. The synthetic sludge without PAM did not present signals related to high molar masses.

**Figure 7:**
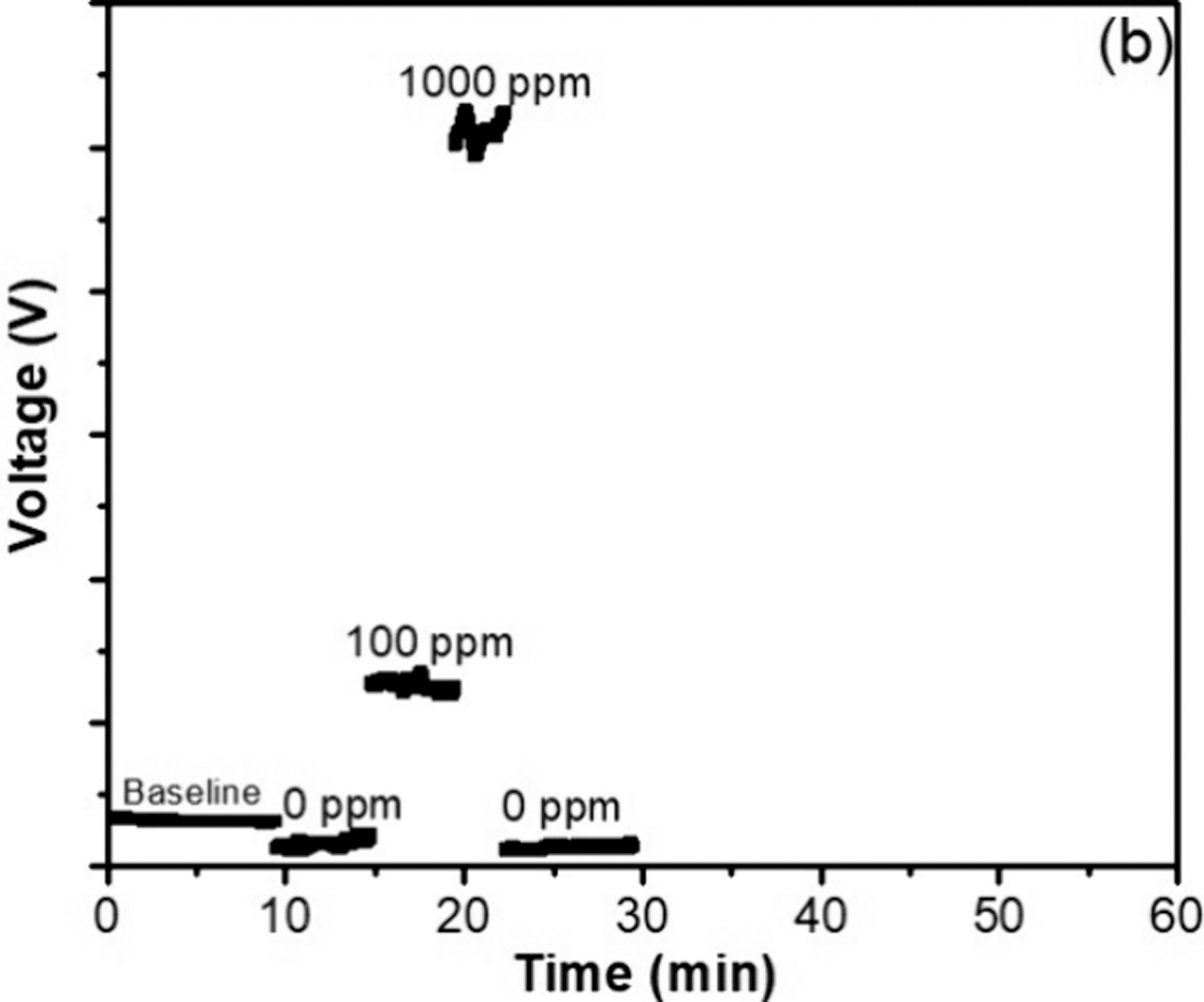
MALS signals of water-soluble compounds recovered from the synthetic sludges containing 0, 100, and 1000 ppm PAM.

### 3.4 Analyses of the Water-Soluble Compounds Obtained by the Residue of the Real Sludge Samples

The presence of PAM was detected in all real sludge samples (Table 4) at different concentrations. As previously discussed, the concentrations were not determined by a quantitative method; therefore, the values are estimated only. Moreover, the concentrations estimated for WPL3 and WPL7 were out of the range covered by the curve obtained with PAM solutions (Figure 2).

**Table 4:** Estimated Concentration of PAM in Real Sludges

**sludge**	**estimated concentration (ppm)**
WPL1	320 ± 2
WPL2	64 ± 3
WPL3	3000 ± 330
WPL4	121 ± 16
WPL5	70 ± 3
WPL6	1260^a^
WPL7	17 ± 2

a Duplicate analysis was not done.

The same solutions were analyzed by MALS, and the results confirmed the presence of the polymer in all sludge samples because the equipment detected material presenting average molar masses of around 10^7^ g/mol.

Figure 8 summarizes the entire procedure used to identify the presence of the polymer in the sludge and estimate its concentration.

**Figure 8:**
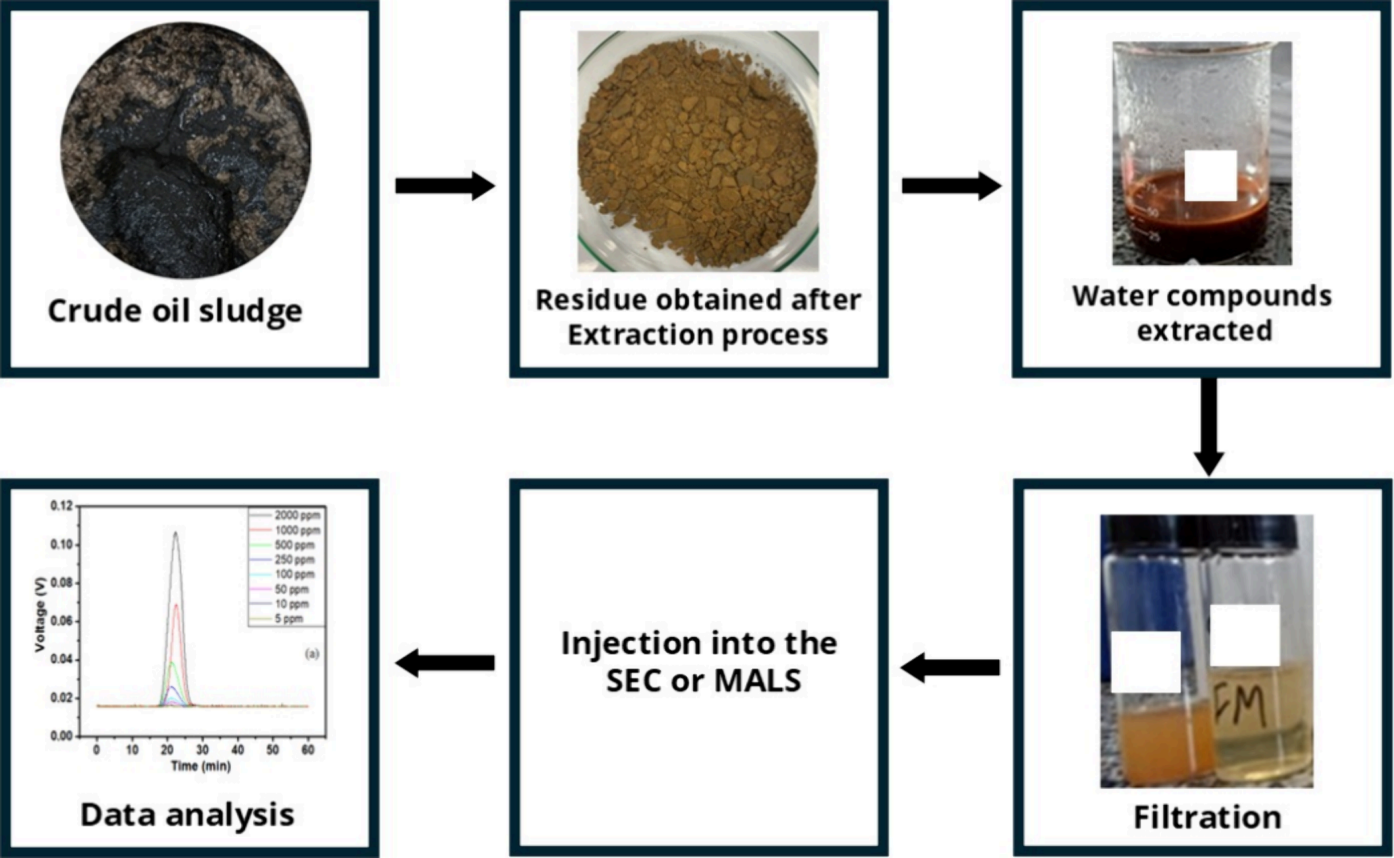
Flowchart of the procedure used to identify the presence of the polymer in the sludge.

## 4 Conclusions

A consistent methodology for polymer (PAM) identification in sludges was developed using 5 synthetic sludges, in which different concentrations of PAM (between 100 and 2000 ppm) were added during the preparation, and this methodology was validated using 7 residues of real sludges (WPL1, WPL2, WPL3, WPL4, WPL5, WPL6, and WPL7) that were withdrawn from the oil industry. Initially, a calibration was carried out in SEC and MALS using different concentrations of PAM (from 5 to 2000 ppm) in brine, where it was observed that the concentrations of 5 and 10 ppm had to be discarded because these were near the baseline. After the methodology determination using synthetic sludges, the aqueous extracted residues of real sludges were injected into the SEC and MALS equipment, and it was observed that most of the sludges did not contain a large amount of polymer, except for the extracts obtained by WPL5 and WPL7 sludges, with 3000 and 1260 ppm of polymer in sludges, respectively. Although the estimated concentrations are semiquantitative, the results obtained for these last two samples mentioned are considered high since the concentration range of 500–2000 ppm is normally used in EOR treatment.
